# A Multidimensional, Multisensory and Comprehensive Rehabilitation Intervention to Improve Spatial Functioning in the Visually Impaired Child: A Community Case Study

**DOI:** 10.3389/fnins.2020.00768

**Published:** 2020-07-24

**Authors:** Federica Morelli, Giorgia Aprile, Giulia Cappagli, Antonella Luparia, Francesco Decortes, Monica Gori, Sabrina Signorini

**Affiliations:** ^1^Center of Child Neuro-Ophthalmology, IRCCS, Mondino Foundation, Pavia, Italy; ^2^Unit for Visually Impaired People, Istituto Italiano di Tecnologia, Genova, Italy

**Keywords:** visually impaired children, multisensory, rehabilitation, development, cognition, spatial cognition

## Abstract

Congenital visual impairment may have a negative impact on spatial abilities and result in severe delays in perceptual, social, motor, and cognitive skills across life span. Despite several evidences have highlighted the need for an early introduction of re-habilitation interventions, such interventions are rarely adapted to children’s visual capabilities and very few studies have been conducted to assess their long-term efficacy. In this work, we present a case study of a visually impaired child enrolled in a newly developed re-habilitation intervention aimed at improving the overall development through the diversification of re-habilitation activities based on visual potential and developmental profile, with a focus on spatial functioning. We argue that intervention for visually impaired children should be (a) adapted to their visual capabilities, in order to increase re-habilitation outcomes, (b) multi-interdisciplinary and multidimensional, to improve adaptive abilities across development, (c) multisensory, to promote the integration of different perceptual information coming from the environment.

## Introduction

Spatial cognition is a multifaceted concept that involves a variety of skills based on the acquisition of knowledge about spatial relationships among entities in the surrounding environment. More specifically, it entails the ability to understand and internalize the representation of the structure, entities, and relations of space with respect to one’s own body (Thinus-Blanc and Gaunet, 1997; Vasilyeva and Lourenco, 2010). The development of spatial competence is essential for perceptual, motor and cognitive development (Newcombe and Huttenlocher, 2000; Newcombe and Learmonth, 2009; Vasilyeva and Lourenco, 2012) and the construction of our own identity (Proulx et al., 2016). Indeed spatial functioning is crucial not only for activities such as localizing stimuli and navigating in the environment, but also for cognitive skills such as perspective-taking, and provides an essential foundation for everyday functioning (Newcombe and Learmonth, 2009; Vasilyeva and Lourenco, 2012).

Studies showed that many spatial abilities (i.e., localization) develop in the very first months of life and are heavily influenced by sensory experience (Gavin et al., 2011). For instance, there is evidence that vision plays a pivotal role in spatial development (Thinus-Blanc and Gaunet, 1997; Eimer, 2004). Therefore, one prediction can be that early visual deficit may interfere with different aspects of psychomotor competence and spatial functioning (Reynell, 1978; Elisa et al., 2002; Dale et al., 2017). Even though some studies reported no noticeable differences in terms of spatial performance between sighted and blind people (Ashmead et al., 1998; Ittyerah et al., 2007), other studies indicate that visual experience significantly influences the acquisition of spatial competence (Cattaneo et al., 2008). For instance, several works have shown that visually impaired people may manifest deficits in specific spatial skills (Cattaneo et al., 2008; Koustriava and Papadopoulos, 2012; Pasqualotto and Proulx, 2012), especially when the visual disability is congenital and results in a total loss of visual acuity (Ellemberg et al., 1999; Maurer et al., 2007; Hadad et al., 2015; Hadad et al., 2017). Moreover, such spatial impairments can have life-long implications, negatively impacting on independent mobility and/or social and work inclusion (Shah et al., 2020). For example, visually impaired people can manifest difficulties in assuming different perspectives, understanding other people’s mental states and emotions, judging others’ trustworthiness (Ferrari et al., 2017), even if mixed results are reported in literature (Bednya et al., 2009; Ma and Han, 2011). It is well known that children with a visual disability may manifest a lack of “initiative” due to the absence of visual feedback emerging primarily at the motor level (Adelson and Fraiberg, 1974; Elisa et al., 2002), then also affecting communication, relational (Fazzi et al., 2007; Fazzi et al., 2019; Chokron et al., 2020) and cognitive levels (Fraiberg, 1968; Fraiberg et al., 1996). For instance, during typical development locomotion facilitates the acquisition of constancy of objects through the visual experience of spatial concepts such as orientation and perspective (Bigelow, 1992). Instead, motor development might be delayed in visually impaired children due on one hand to the difficulty of conceiving an object as existing in space, since for the blind child the ability to search for an object precedes and facilitates locomotor development (Fraiberg et al., 1996; Fazzi et al., 2011; Papadopoulos and Koustriava, 2011) and, on the other hand, to the lack of the physiological feedback to the vestibular and proprioceptive system mediated by vision (Prechtl et al., 2001). Overall, such behavioral findings indicate that visual impairment may have a negative impact on psychomotor and spatial development and result in severe delays in adaptive abilities across childhood. Also, some neurophysiological studies showed that a reduced visual input significantly impacts the functional organization of the cortical visual system during infancy (Hubel and Wiesel, 1977; Blakemore, 1991; Price et al., 1994), supporting the general view that visual experience might be important for perceptual and cognitive development. In the absence or reduction of vision, an early intervention appears to be necessary to foster overall development and encourage independence and social inclusion. This can be made with the involvement of a multi-disciplinary professional team and the direct engagement of caregivers, thus supporting the child not only in the healthcare setting but also in the various contexts of life (Rainey et al., 2014).

## Background and Rationale

The World Health Organization (World Health Organization, 2017) estimated that in 2015 252.6 million people worldwide were visually impaired, of whom 36 million were classified as blind, with an estimate of 19 million children below the age of 15 years were visually impaired (1% of the total population in this age group), of whom 1.4 million had irreversible blindness (0.08% of the total population in this age group).

In the previous paragraph, we highlighted that visually impaired people can manifest difficulties in the development of spatial competence (Cattaneo et al., 2008; Koustriava and Papadopoulos, 2012; Pasqualotto and Proulx, 2012), especially when visual experience is compromised from birth (Ellemberg et al., 1999; Maurer et al., 2007; Hadad et al., 2015; Hadad et al., 2017). Even though the visual system influences significantly spatial information supplied by other modalities (Spence and Driver, 2012), spatial knowledge relies also on other sensory modalities such as touch, proprioception, kinesthesia, and audition (Millar, 2012). Such findings would argue in favor of early adoption of integrated intervention strategies when dealing with a congenital visual impairment, to promote perceptual and cognitive development and also social cognition through multisensory activities (Berardi et al., 2015; Purpura et al., 2017). Indeed, training intact sensory modalities such as audition and touch from an early age is essential to help the child building a relation with the environment and dialoguing with caregivers and peers. Recent evidence have shown that multisensory experiences such as audio-motor training activities performed from an early age can support the development of spatial abilities in the visually impaired child (Cappagli et al., 2017b, 2019). Moreover, other evidence demonstrated that early non-visual spatial experiences can influence spatial acuity in visually impaired people: more specifically, the earlier children start an orientation and mobility training, the more accurate their space perception is across life-span (Fiehler et al., 2009). Finally, a growing body of literature has shown that echolocation, namely the ability to orient in space by relying on self-produced echoes, may improve the general sense of auditory space in blind people (Kolarik et al., 2014; Vercillo et al., 2014), suggesting that spatial competence can be acquired through alternative non-visual senses. In this regard, a recent article (Norman and Thaler, 2019) supports this view by demonstrating that in blind echolocators the functional topography of the occipital cortex is used to map sensory input from an atypical modality for a directly analogous task-specific purpose (sound localization).

Nonetheless, a very recent review (Elsman et al., 2019) revealed that rehabilitation interventions for the visually impaired are rarely adapted to children’s visual capabilities and very few studies have been conducted to assess their short-term and long-term efficacy. Therefore, there is the need to determine which interventions are effective and evaluate their effectiveness to increase functioning, participation, and quality of life in visually impaired children. We argue that the lack of homogeneous results on rehabilitation techniques is due not only to the use of a variety of outcome measures (many of which were not specifically developed for children with visual impairment) but also to the implementation of rehabilitation programs that do not differentiate intervention activities based on the visual and developmental profile of children. Taking into consideration the nature and the degree of the visual disability as well as the developmental profile and additional disabilities would result in an individualized therapeutic approach aimed at boosting perceptual, motor, cognitive, and socio-emotional potentials from an early age across different contexts of everyday life (Sonksen, 1997).

With this aim, we propose an integrated model of intervention that is: (a) multi-interdisciplinary, because it is based on the contribution of different professional figures (child neuropsychiatrists, neuropsychomotor therapists, ophthalmologists, orthoptists, psychologists, speech therapists, orientation and mobility trainers); (b) multisensory, because it proposes activities encouraging visually impaired children to integrate different perceptual information coming from the environment; (c) individualized, because activities are based on the visual and developmental profile of child; (d) multidimensional, because re-habilitation goals rely on a parallel collaboration of professionals and caregivers in the different contexts of life. The proposed intervention, in the context of overall development promotion, is intended to train spatial abilities as well as perceptual, motor, relational, and cognitive abilities linked to the acquisition of spatial competence, by assuming that the latter drives the acquisition of fundamental high-order skills such as perspective taking and problem-solving (Newcombe and Learmonth, 2009).

## Methodological Aspects

In this work, we describe a re-habilitation intervention characterized by its diversification based on the visual and developmental profile by reporting the case of M., a visually impaired child enrolled in this approach for ten years (June 2009–June 2019), from the age of 9 months. This work has been carried out at the Center of Child Neurophthalmology of the IRCCS C. Mondino Foundation of Pavia, a national reference clinic for the diagnosis of visual disturbances and the re-habilitation of the child with visual impairment.

### Targeted Population

**FIGURE 1:**
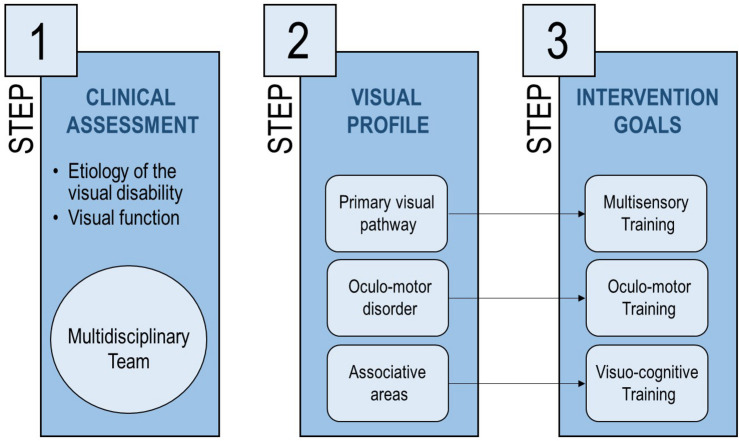
The interventional approach proposed in the current manuscript, described in three steps aiming at identifying the most suitable rehabilitation approach based on visual deficit.

The Center of Child Neurophthalmology of the IRCCS C. Mondino Foundation (Pavia, Italy) deals with different types of visual disorders, from both a diagnostic and re-habilitative perspective. Our re-habilitation intervention is based on three steps (Figure 1):

1. At first admission, a comprehensive clinical assessment is performed to identify the clinical profile of the patient (etiology of the visual disability, functional vision, developmental profile, and neuropsychiatric aspects);
2. Depending on the clinical profile of the child, the most damaged visual subsystem is defined: (a) primary visual pathway, affecting visual perception (e.g., in retinal dystrophies), (b) oculo-motor system, conditioning ocular motility (e.g., in oculo-motor apraxia and nystagmus), (c) associative visual areas, regarding visuo-cognitive skills (e.g., in cerebral palsy);
3. Re-habilitation goals and strategies are defined considering the most damaged visual subsystem and the developmental profile of the patient, together with possible comorbidities.

### Case Study

We present the case of M., a child affected by a congenital disorder of the primary visual pathway, specifically retinal dystrophy (Leber Congenital Amaurosis), diagnosed at the age of 5 months on the basis of a poor vision from birth, abnormal eye movements, macular atrophy attenuated retinal vessels, and severely reduced scotopic and photopic electroretinogram and abnormal Visual Evoked Potentials (De Laey’s criteria; Fazzi et al., 2003). He was enrolled in our re-habilitation intervention since his first admission at our clinic (9 months of age). At admission, we performed clinical and instrumental evaluations (i.e., electrophysiological exams, EEG, and brain MRI) to specifically define the visual impairment and investigate possible comorbidities and syndromic forms of retinal dystrophy. Neurophthalmological examination showed sluggish pupillary reactions, nystagmus, roving eye movements, and a deficit of fixation and pursuit that improved with the addition of sound; fundus oculi examination confirmed the presence of macular atrophy and attenuated retinal vessels; no refractive errors were also reported. Binocular grating acuity (Teller Acuity Cards (Teller et al., 1986)) testable only at the distance of 38 cm, was of 0.60 cy/deg, revealing severe perceptual deficit with residual close-up visual acuity; contrast sensitivity, evaluated with Hiding Heidi Low Contrast Face Test (Chen and Mohamed, 2003), was also altered (close-up response only for high contrast stimuli). Oculo-digital signs such as “eye-pressing” were present. Central Nervous System involvement was excluded, along with other comorbidities. A panel gene testing confirmed the diagnosis of inherited congenital non-syndromic retinal dystrophy involving NMNAT1 gene.

Neurological examination was normal except for mild aspecific hypotonia, frequently described in severely visually impaired children (Fazzi et al., 2005b). The child had good relational competences and attention span for his age, with some degree of emotional stress, expressed through motor hyperactivity and emotional lability, probably due to the necessity to adapt to the environmental requests. Head control and sitting position were acquired, rolling over was rarely observed and improved with the aid of auditory stimulus. Reaching and grasping of objects were performed only with audio-tactile integration, and subsequent occasional integration of visual information. The child functionally used both hands and showed a preference for specific textures. Neuropsychomotor development, evaluated by Reynell-Zinkin Scale (RZS) (Reynell, 1978), was characterized by a slight decline in the area of environment exploration and expressive language.

### Re-habilitation Strategies and Results

**FIGURE 2:**
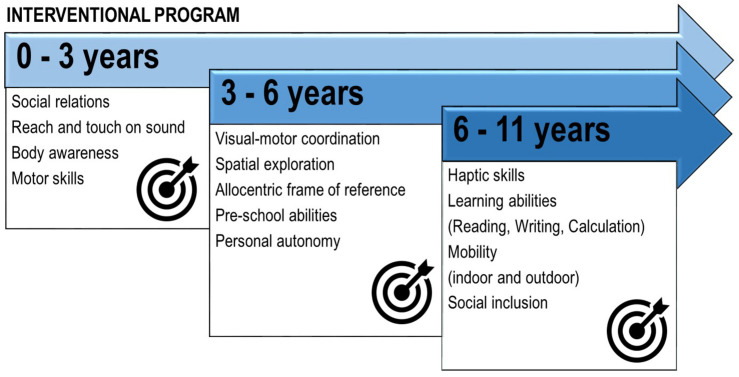
Summary of the main rehabilitation goals for the patient M., affected by a deficit in the primary visual pathway with residual close-up visual acuity, according to his chronological age.

**FIGURE 3:**
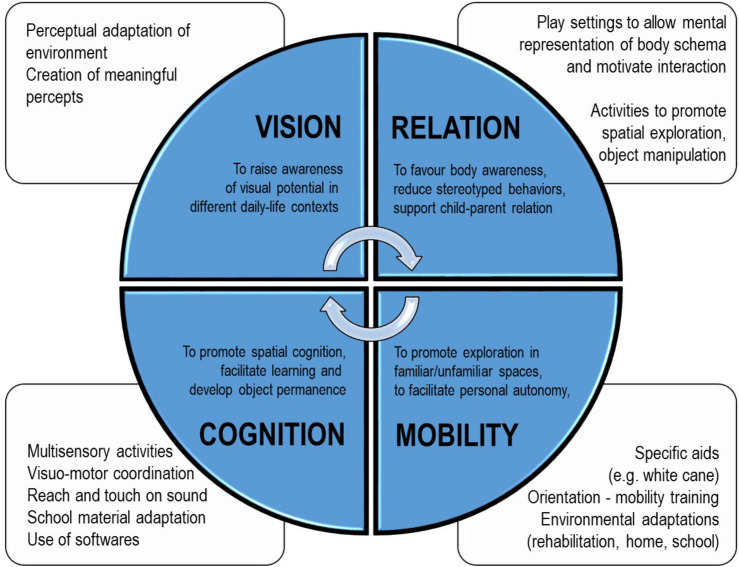
Summary of the main rehabilitation activities for each domain considered (vision, cognition, mobility, and relation) for the patient M., affected by a deficit in the primary visual pathway with residual close-up visual acuity.

Based on clinical assessment, the re-habilitation intervention was focused on the observed developmental fragilities and visual profile (see Figure 2 for a general description of re-habilitation goals, Figure 3 for a more specific description of intervention strategies and Table 1 for a detailed description of re-habilitation activities based on age and developmental goals). During all the re-habilitation process, the intervention also considered the child’s developmental profile and the main difficulties that a visually impaired child can encounter compared to sighted children, according to the literature on both typical and visually impaired children development and relying on our experience (Adelson and Fraiberg, 1974; Fraiberg et al., 1996; Cappagli and Gori, 2016; Vercillo et al., 2016; Cappagli et al., 2017a; Fazzi et al., 2010). We will illustrate the case study by presenting the main re-habilitation strategies and results for each developmental stage (9 months to 3 years; 3 to 6 years; 6 to 11 years) defined as “critical windows” for acquiring fundamental perceptual and cognitive abilities in the typical development (see Figure 2 and Table 1).

**TABLE 1:** Re-habilitation activities performed according to developmental age (0–3 years old, 3–6 years old, and 6–11 years old) and perceptual and cognitive domains.

Age	Developmental goal	Re-habilitation activities
0–3	**Functional vision and multisensoriality** Promotion of body awareness and functional use of sensory modalities	• Visual environmental adaptations (e.g., setting with highly contrasting colors and illuminated objects) • Multimodal inputs (objects with emphasized visual, tactile and sonorous features, such as balls with a bell inside, drums, soft puppets made of different cloths)
	**Socio-emotional cognition** Promotion of parent–child relationship, communication, functional use of language and reduction of stereotyped behaviors, motivation to explore the external world	• Creation of a play environment with the parent who acts as a “mediator” in all the activities • Games based on physical contact and vocal communication to recognize emotions • Tactile exploration of the parent’s face (discrimination and identification of the different parts, also through residual vision) • Interactive games with the parents (e.g., the child and the parent/therapist have to exchange an object after naming it; the parent/therapist presents objects by using alternative sensory channels (sound and touch) • Symbolic play (e.g., through interactive invention of short stories, symbolic use of objects)
	**Sense of self** Promotion of proprioception as baseline for motor development and construction of bodily self	• Play activities in which the parent/therapist positions a vibrating or sonorous object on a visible (e.g., hand) or not visible (e.g., neck) body segment of the child and the child is asked to find and remove it • Denomination activities in which the child is asked to name the body part on which a vibrating or sonorous objcet is placed, in order to develop verbal knowledge of different body parts (the activity can become reciprocal with the child positioning the object on a parent’s body segment)
	**Cognition (including spatial cognition)** Promotion of sensorimotor intelligence, reach and touch on sound, object permanence, mental imagery	• Activities in which an object is only presented by sound or touch, motivating the child to reach and grasp it • Gradual exposure to different objects with integrated use of sight (if possible), touch (through bimanual exploration) and hearing: ∘ objects commonly used in everyday life ∘ objects with specific or peculiar audio-tactile characteristics ∘ bi-dimensional and tri-dimensional objects with different shape, texture, dimension, weight and presented at progressively greater distances and in different spatial positions • Games based on picture puzzles construction in which pieces are actively searched in a visually adapted environment • Exposition to spatial language
	**Motor development** Promotion of gross-motor (postural control and crawling, walking) and fine-motor (grasping and manipulating abilities) functions	• Perceptually adapted (visually contrasted, multisensory) and spatially organized (safe and circumscribed) environments: the play corner is characterized by sonorous-tactile elements (e.g., tactile tiles and differently textured cloth), delimited by pillows or smooth, soft furniture • Activities in which the child is motivated to reach lights/shapes/sounds on colorful panels on a wall or has to search objects guided by the voice of the parent, promoting independent exploration while keeping a distance from the caregiver
3–6	**Spatial cognition and visuo-motor coordination**	• Praxic-construction tasks with adapted materials (e.g., recreation of a shape by assembling the pieces in which the shape was cut, 2D and 3D puzzles, tangrams) • Block design: free and structured (i.e., from a model) assembling and deconstruction, during storytelling activities • Play activities in which the child has to place small objects in a line or create a geometrical shape • Training of spatial transformation with ego- and then allocentric frame of reference (e.g., reproduction of a configuration of objects on a board by assuming different spatial positions) • Training on opposite spatial concepts (on top of/under, tall/short, in front of/behind) applied both in relation to the child’s position and to the objects’ positions (for example, the child has to find a ringing object on his right, and then to move and stop in front of the object) • Training on visuo-spatial exploration: identify differences in the spatial layout of similar images, analyze the details in a figure, recognize different orientations of lines • Comprehension of auditory stories based on topographical information • Visual and visuo-tactile (with contrasted and embossed materials) activities with bi-dimensional and tri-dimensional objects in order to foster the development of perceptual and cognitive abilities such as: • topographical reasoning • spatial orientation • reproduction of 3D, 2D, and graphic models • Software based on visual and auditory inputs
	**Pre-school abilities** Promotion of visuo-cognitive skills, memory, sustained attention	• Visual and visuo-tactile (with contrasted and embossed materials) activities with bi-dimensional and tri-dimensional objects in order to foster the development of perceptual and cognitive abilities such as: • bimanual exploration of objects • recognition/detail analysis • semantic categorization • drawing activities
		• Auditory activities in order to reinforce mnestic and attentional skills related to sonorous stimuli in the environment: • detection (e.g., listening to sounds and then verbalizing the number of sounds) • discrimination (e.g., distinguish different sounds presented together)
		• memory (e.g., auditory memory game) • identification (e.g., listening to a sequence of sounds or words and verbalize the listening order) • Material adaptations (highly contrasted paper sheets, with well-defined margins and tactile references to help spatial organization of the sheet)
	**Mobility and personal autonomy** Promotion of the ability to move around and be autonomous in everyday routines to improve social inclusion	• Environmental adaptations (in the training setting, at home, at school, if possible) • Motor activities based on tactile and/or sonorous references to improve walking fluidity and speed • Motor training based on identification of position landmarks in a natural environment and construction of the first cognitive maps • Introduction of self-protection techniques (pre-cane) and use of external reference points to orient in novel environments (e.g., windows, lights, and doors) • Promotion of music and sports with peers
6–11	**Haptic and visuo-cognitive skills** Promotion of visuo-tactile integration, tactile discrimination	• Activities with the use of tiflologic cards with embossed spacing and the use of an awl to train the ability to organize points in the space and pursue a gradual exposure to Braille code
	**Spatial cognition** Promotion of the ability to switch from egocentric to allocentric frames of reference in different contexts	• Prosecution of the activities described for age 3-6 with progressively more complex activities and requests • Spatial and topological organization tasks • Visual-spatial training with exercises based on translations, rotations, overturning of geometric and plane figures
	**Learning abilities** Promotion of school inclusion and learning	• Environmental adaptations (illumination, first-line lifted desk, and bookrest) • Adapted materials integrating visual and tactile features: graphic cards with enlarged numbers and letters, high target-background contrast, thick margins • Specific cross-modal software for reading and writing (e.g., training of decoding abilities, accuracy, and speed through tachistoscopic presentation of single words and timed reading of brief texts) • Specific cross-modal software for calculation, geometry, auditory attention and memory (e.g., sound detection, discrimination and identification activities, auditory memory games, listening to texts followed by a quiz on the text content) • Provision and training of low vision aids: ∘ digital audio-books ∘ computer screen reading software ∘ tablets computer-based Assistive Technologies (with applications such as screen magnifiers, optical character recognition and text-to-speech conversion) • Teaching of braille code • Compensatory tools (personalized reading and writing materials, such as notebooks with embossed margins and spacing, textbooks with clear and dimensionally adapted letters and line-spacing) • Dispensatory strategies (avoid copying from blackboard, use of capital letters only)
	**Mobility, personal autonomy, and social inclusion** Promotion of the ability to move indoor and outdoor and acquisition of personal autonomy and activities with peers	• Training in the use of self-protection techniques (introduction of white cane) • Map design: training in the memorization of previously performed routes which are verbalized and then redesigned on a rubber surface with an awl • Training in adaptive abilities for everyday life (e.g., to get dressed properly, to prepare school backpack, to use a phone…) in order to spend more time at school and/or with peers • Promotion of music and sports with peers

Training sessions took place twice a week until 3 years of age, once per week in the 3–6 years old period, and twice per month during school age. A periodic neurological and developmental evaluation (every 6–8 months) was performed to tailor re-habilitation activities step-by-step, based both on “standardized” methods and on qualitative observations mainly focusing on the most frequently impaired domains in visually impaired children (e.g., motor initiative, relation, and expressive language). Our comprehensive assessment, both from a qualitative and quantitative perspective, included socio-emotional, relational, and adaptive skills. At the beginning of the intervention and until 3 years of age, a standardized evaluation was made with RZS (Reynell and Zinkin, 1975), a developmental tool for visually impaired children; when the child grew older and was able to sustain more structured evaluations, cognitive evaluations were performed, generally once per year, and based on available standardized tests, such as Wechsler Scales (Wechsler, 2002; Vaughn-Blount et al., 2011); other tests used to evaluate specific domains were the NEPSY-II (Brooks et al., 2010), the Developmental Test of Visual Perception (Brown and Hockey, 2013), batteries to assess learning skills (specifically developed for Italian population), and other non-standardized tests such as topological, figure recognition, and categorization tasks created and performed by our therapists. Results were periodically gathered and discussed in team meetings in order to evaluate the outcome and define the goals of future intervention.

A fundamental aspect of our re-habilitation approach is that it entails activities related not only to the clinical setting but also to the home setting, introducing environmental adaptations transposed in the child’s everyday environments, from the perspective of a multidimensional approach. Psychological support for parents is proposed, focused on the acceptance of child’s disability and the promotion of the ability to understand his needs and his modalities of expression (e.g., signs of emotional distress expressed with stereotyped behaviors and oculo-digital signs), to support the parent–child relationship. Parents are also asked to actively participate in the training sessions so that they can see the activities and be explained how to talk and play with the child in different contexts. For example, they are taught to interact with the child through physical contact and vocal communication, respect the time the child needs in the mutual interactions and in response to specific requests, give the right level and kind of input. Specific games based on sound and touch are showed to the parents so that they can reproduce them at home, together with environmental adaptations (see Table 1). When the child begins to spend a large amount of time at school, teachers are involved in the re-habilitation process as well, with periodic meetings to discuss the best strategies to promote social inclusion and learning.

### From 9 Months to 3 Years of Age

#### Strategies

The first step of the intervention was directed at promoting sensorial experience and overall development, with a focus on relational, neuromotor and cognitive aspects. These goals were pursued with the creation of highly “socializing” play settings, in which activities based on the use of the voice or tactile perception or emphasizing visual information regarding human face were proposed. Parents were trained to establish a dialogue with the child, using different sensory inputs and catching signs of emotional distress which could interfere with the relational and spatial experience, making object relations less meaningful. The environment was perceptually adapted with the introduction of sensorial panels and audio-tactile objects so that the child could become more conscious of the environment itself and motivated to move and explore his personal (space occupied by the body) (Vaishnavi et al., 1999) and peri-personal (space surrounding our body within the reach of our limbs) (Làdavas et al., 1998) dimensions. Particular attention was dedicated to the ability to locate and grasp an object after sonorous/tactile input and the acquisition of the object permanence: this competence, known as “Reach and touch on sound” (Fazzi et al., 2011), seems to serve also as an organizer of gross-motor experience. The use of chromatic contrasts and lively colors helped the child to become aware of his residual visual function and optimize its integrated use with the other sensory modalities. Particular attention was dedicated to sensory inputs beneficial to the construction of body schema, intended as an on-line representation of the body in terms of posture and its extension in space (Head and Holmes, 1911; Holmes and Spence, 2004). For example, one of the activities proposed was based on the physical contact of audio-tactile objects on various parts of the child’s body associated with the denomination of the body segment; the child was asked to search the object on his body and on the parent’s body, during simple reciprocal activities (see Table 1, “Socio-emotional cognition” and “Sense of self”).

At the age of 18 months, after almost 1 year of treatment, we noticed a positive change in awareness and integrated use of different sensory modalities, better functional use of exploration strategies with a prompt ability to find objects and people in the environment and a slight improvement in binocular grating acuity evaluated by Teller Acuity Cards (4.7 cy/deg) at the same distance of 38 cm. Altered contrast sensitivity was confirmed by Hiding Heidi Low Contrast Face Test (high contrast stimuli perception only); no refractive errors were reported. Also, social participation and communicative intentionality were improved, as shown by the reduction of restlessness and oculo-digital signs. Some degree of inattention was still observed during tasks requiring prolonged listening or use of visual-tactile information. RZS confirmed a slight decline in the sensory-motor and language areas but a developmental setback was not observed. From 18 to 36 months of age, the intervention focused on the improvement in the functional use of haptic information and on object manipulation, in order to strengthen exploration, recognition, sensorial semantic categorization, and topological relationships. We then proposed activities of haptic exploration to discriminate different textures (smooth/rough, stiff/soft, etc.), shapes (square, circle, etc.), dimensions, weights, and other physical attributes of objects. Play settings were adapted to be spatially organized and present deep symbolic meaning: we included objects with a meaning for the child to help him recognize them and motivate him to use them in a functional way, possibly fostering the parent-child relationship. In an interactive and entertaining setting, active exploration was stimulated with the use of real objects, placed in different space plans, and through verbal guidance also exposing the child to spatial language (e.g., spatial location words like “up” or “down,” deictic terms like “here” or “there,” dimensions, shape terms, spatial orientations, etc.). This use of language seems to help to elicit more spatial language production and to build later skills such as the ability to do spatial transformations and analogies (Verdine et al., 2019) and it is recommended also in parent-child interactions. Playing with toys that incorporate shapes (e.g., shape sorters), labeling them, and discussing shape properties may be among the earliest spatial experiences parents provide (Verdine et al., 2016).

#### Results

After 1 year of training, clinical observation revealed a positive change in the child’s close-up visual and behavioral performances, at least partially due to the physiological maturation of the visual system, sustained by an adaptation of the sensory experience to foster the use of residual visual function. Above all, we observed improvement in ocular motility and coordination, improvement in the functional residual vision and good abilities to locate visual targets even in the absence of sound-tactile facilitation in a visually adapted environment (e.g., with the use of highly contrasted patterned panels), and at a near distance. M. used these skills to explore the surrounding environment functionally: in particular, crawling and postural passages were easier when the space of action was reduced and perceptually adapted. Furthermore, residual vision was functionally used even in grasping: after locating objects by relying on the visual feedback, he integrated various sensory information (sound, touch, and sight) to explore them. We also noticed a good progression in gross motor function, and the motor milestones (i.e., standing and walking) were reached as expected compared to sighted peers.

### From 3 to 6 Years of Age

#### Strategies

In this phase, intervention was particularly focused on the promotion of autonomy and the acquisition of pre-school abilities, with specific attention to visual-motor coordination and spatial exploration. When M. was four years old, some difficulties emerged involving visuo-tactile and visuo-motor integration, probably reflecting impairments in visual monitoring and fatigability in the use of visual information, which caused delays in the development of fine-motor skills. The lack of visual-motor integration also negatively interfered with the activities of daily living, making it difficult to acquire personal autonomies. The re-habilitation approach was then readapted to the child’s needs and the main goal in this phase was to strengthen different sensorial functions to sustain cognition and learning and to promote autonomy through orientation and mobility training in broader spaces, also with the development of social skills as an endpoint (see Table 1). The main strategy to reach these goals was the training of multisensory integration. We trained auditory attention through activities of detection (e.g., asking the child to pay attention to and verbalize the number of sounds presented), discrimination (e.g., asking the child to distinguish different sounds of increasing number and complexity, also using a sort of “memory” game with sounds), identification (e.g., inviting the child to listen to a sequence of sounds and/or words and verbalize the type of the stimuli). Some activities were based on the integration of visual and tactile exploration, to train recognition, association, categorization abilities and learn spatial relations (topological and topographic) and action planning, always sustained by the therapist-use of language to mediate knowledge. These visual-tactile spatial tasks were particularly useful for the future learning of braille code, geometric and graphic abilities. Some cognition enhancement games were also used, based on logic and short stories listening and comprehension tasks. From the perspective of a multidimensional approach, an important aspect of intervention in this phase was the introduction of devices and strategies to adapt the environment and material to the sensory characteristics of the child (for example, the use of a reading desk, the spatial organization of paper also with the use of tactile marks) during graphic tasks at home and/or at kindergarten. Training on orientation and mobility was also introduced. This training was based both on a play setting with the therapist and on the intervention of a personal autonomy instructor focusing on daily life necessities. The general aim was to improve the ability to locate objects in the space by using the child’s body as a reference (for example, asking the child to take the ringing object at his right) and then using the surrounding space as reference (i.e., asking the child to move and stop in front/behind the object). The environment was adapted by creating spatial paths with tactile, visual and/or sonorous landmarks. Autonomy and adaptive skills were promoted by training the child to recognize and locate landmarks in the natural environments and to use protection techniques, such as the use of a pre-cane in wide and crowded places.

#### Results

At three and a half years old, the child used and integrated visual information with auditory and tactile information for the exploration of the environment and/or objects and located visual targets in the peri-personal space even in the absence of audio/tactile facilitation. This visual competence was used effectively by the child to direct his movements in space. In the exploration and knowledge of close objects, we observed an improved functional use of touch: he showed good skills in bimanual coordination and systematic exploration both to recognize and discriminate the shapes and structures of objects and to analyze topological relationships between them. The overall positive evolution was confirmed by Wechsler Primary and Preschool Intelligence Scale (WPPSI-III) where M. reached a verbal index of 131 and performance index of 106 (total intelligence quotient: 122), showing an adequate spatial competence. The same competences were also measured qualitatively: the child manifested good capabilities of exploring small and familiar environments by using landmark location abilities (for example, the lights coming from windows) and memory skills. Neuro-visual examination showed the persistence of roving eye movements and nystagmus, poor visual fixation and discontinuous smooth pursuit at near. Testing with LEA single symbols (Hyvärinen et al., 1980) confirmed severe low vision (2/10 for near distance, no answers for far distance); other findings were altered contrast sensitivity (evaluated with Hiding Heidi Low Contrast Face Test and LEA symbols) and color perception (Color Vision Test Plates For The Infants) (Lee et al., 1997), and absent stereopsis (Lang Stereotest) (Lang and Lang, 1988); visual field was clinically difficult to evaluate also for his age. Appropriate lenses were prescribed for the evidence of a refractive error (hypermetropia and astigmatism). At 6 years of age, continuous improvements were observed within psychomotor development. Concerning the posturo-motor organization, the ability to walk, run, go up and downstairs and perform postural passages with good motor fluidity and autonomy was acquired. He acquired the ability to move in unknown environments, preferentially using auditory information (i.e., adult’s voice) as a guide, although he also used visual information (visual location) functionally, using spatial landmarks such as light points and/or bright color furnishings. Manual organization in bimanual tasks also appeared well modulated even though slow.

### From 6 to 11 Years of Age

#### Strategies

In this phase, the intervention had the main goal of developing and sustaining reading, writing, geometry and math skills together with personal autonomy. Re-habilitation was then focused on supporting visual-spatial perception connected with visuo-cognitive skills (e.g., translations, rotations, and overturning of geometric and plane shapes – see Table 1 for details). Moreover, it was directed on promoting the enhancement of basic reading-writing skills, increasing decoding accuracy and speed through the use of graphic cards with special features (enlarged numbers and letters with high target-background contrast, different materials, thick edges, adapted bookrest), video writing programs with vocal synthesis and iconographic representation, tachistoscopic presentation of single words, timed reading of small passages, and graphic material. Empowerment of functions such as sustained attention, spatial memory both with visual and auditory tasks, and visuo-cognitive abilities was performed through the use of visuo-tactile materials or specific software. Also, activities of sound discrimination and identification were required, along with prolonged listening and auditory memory tasks. At the same time, haptic competence was continuously enhanced through activities of tactile discrimination, categorization, spatial and topological organization and tactile-kinesthetic memory, also to facilitate the learning of the braille code considering that severe low vision remained stable over time. M. successfully learned to use braille, either to read and write (using a typewriter and, subsequently, a computer). Concerning mathematical competence, activities aimed at enhancing the visual-spatial orientations for the correct reading-writing of numbers and signs and an adequate numerical queuing were proposed; for the learning of math, the child used traditional printed, highly contrasted material (black/white) with enlarged numbers and a low-vision calculator. With the collaboration of school operators and the tiflologist, in agreement with the family, we introduced compensatory (personalized reading and writing materials) and dispensatory (e.g., avoid copying from the blackboard, use of capital letters for writing) tools and strategies to facilitate the learning process. At the same time, we proposed activities to foster spatial cognition both in indoor and outdoor environments along with personal autonomy, with a subsequent positive outcome on social aspects. This was done by improving the protection techniques, developing strategies to remember every-day routes (e.g., by redesigning them with the rubber surface after verbalization by the child) and introducing the use of a white cane at the age of 9 years old, as soon as the child was ready to accept and functionally use it.

#### Results

At nine years of age, in the context of visual-tactile exploration used for visuo-cognitive tasks (consistent with the objectives of the re-habilitative intervention implemented since school age), the child was able to complete construction tasks, such as block design, and recognize spatial relationships and orientations. In our opinion, the acquisition of an allocentric frame of reference, according to which locations are described using object-to-object relationships independently from the subject’s point of view (object-centered representations), may represent a sign of good outcome in terms of spatial competence. Allocentric capabilities were trained and evaluated, for example, during tasks in which the child had to reproduce the spatial configuration of textured coins on a board by assuming different spatial positions (see Table 1). Concerning personal autonomies, the child appeared to employ useful strategies to move with bodily awareness in the environment, paying attention to find the visuo-spatial points of reference useful for orientation in different contexts (room, refectory, corridor, and classroom) and to perform more direct spatial paths. Also, personal autonomy and effectiveness in using the white cane to move outdoors improved: the child showed good capabilities of managing to carry out medium-length and complex routes with minimum assistance and good ability to orientate in space.

**FIGURE 4:**
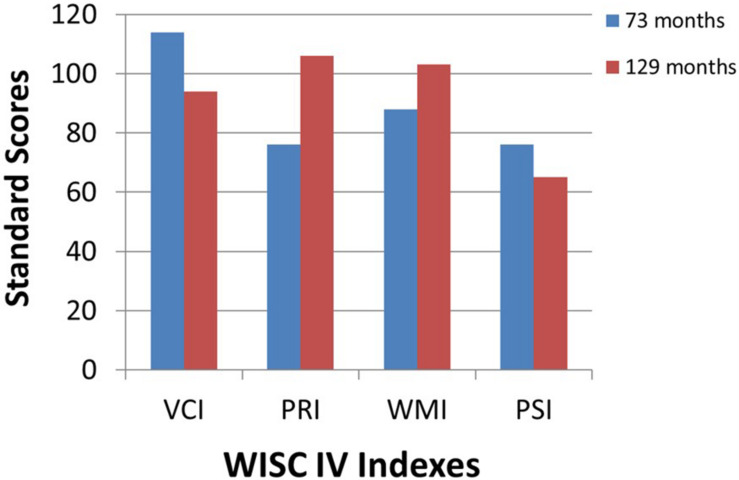
First (73 months) and last (129 months) cognitive assessment of the patient M. with the Wechsler Intelligence Scale for Children (WISC-IV). VCI, Verbal Comprehension Index; PRI, Perceptual Reasoning Index; WMI, Working Memory Index; PSI, Processing Speed Index.

At the time of writing of this work, M. is 11 years old. When he was first tested with Wechsler Intelligence Scale for Children at the age of six years old, his global intelligence quotient was in the range of typical development with a quite disharmonic profile showing adequate scores for verbal and working memory tasks and borderline scores in the Perceptual Reasoning and Processing Speed Index. In the last cognitive evaluation, his profile fitted perfectly in the typical range with a harmonic profile and good results in Perceptual Reasoning Index, demonstrating an improvement in visual-spatial abilities; the decline in Processing Speed Index may be due to fine-motor slowness related to the visual deficit (Figure 4). From the emotional perspective, M. has always shown good coping and relational abilities, and no signs of isolation or passivity have ever been observed.

## Discussion

In the present work, we presented a paradigmatic case of our re-habilitation program based on a multi-interdisciplinary, multidimensional and multisensory approach for children affected by a visual impairment causing difficulties in spatial development. In fact, it is widely accepted that the lack of early visual experience may have a negative impact on the development of spatial abilities as well as motor skills and mobility (Fraiberg, 1968; Morrongiello et al., 1995; Prechtl et al., 2001; Sonksen and Dale, 2007). Moreover, some studies (Aggius-vella et al., 2017; Purpura et al., 2017; Cuppone et al., 2018) showed how the use of other senses may help blind and low vision children to reach developmental milestones which would otherwise be difficult to achieve, such as object permanence (Elisa et al., 2002; Fazzi et al., 2011), which is one of the main goals of our early re-habilitation. With this work, we argue that our approach may facilitate the acquisition of the ability to adapt to environmental requests, particularly important in the context of spatial cognition. Recent studies have confirmed that multisensory re-habilitation approaches may help the child to move independently in the environment and encode spatial and socially relevant information (Aggius-vella et al., 2017; Cappagli et al., 2017b). Moreover, several evidences have demonstrated that multisensory protocols are more effective than training protocols based on unisensory stimulus regimes due to preexisting congruencies of information coming from the different senses (Shams and Seitz, 2008). This is confirmed by studies suggesting that multisensory-integrated re-habilitation methods could be effective for children with sensorial impairment (Purpura et al., 2017). In line with this view, a very recent article has shown that hemianopia can be rehabilitated with an audio-visual training procedure based on spatiotemporal concordant stimuli, stressing the benefits of multisensory stimulation (Dakos et al., 2020). Overall, such findings could be explained in terms of “crossmodal plasticity,” defined as the possibility that sensory deprived regions become responsive to the remaining modalities (in the case of visually deprived people, auditory and tactile modalities) (Dormal et al., 2012) and consequently support the notion that functional specializations of cortical sensory areas is modality-independent. In other words, the positive outcomes of our re-habilitation approach could be at least partially based on the notion that multisensory stimulation (e.g., audio-visual) trains visual cortices to preserve their typical specializations, e.g., to respond to spatial-related stimulation, ultimately further strengthening the emerging proposal that brain organization is driven by specific sensory-independent computations rather than by specific unisensory-inputs as classically conceived (Pascual-Leone et al., 2005; Heimler et al., 2015 Amedi et al., 2017).

Our re-habilitation approach strongly relies on these concepts and adapts to the nature and degree of the child’s visual impairment. In the first months of life, multisensory information (auditory and tactile) enhance the experience and motivation of the child to explore the surrounding space, laying the foundation for the use and training of the other senses to support vision. As reported in some studies (Vercillo et al., 2016; Cappagli et al., 2017b), the use of multisensory experience may help to develop spatial skills that would be otherwise compromised by the lack of visual experience. Also, if a residual visual function is present (as in the case of child M.), visual-haptic and visual-auditory activities may be useful to promote the integration of vision and implement perceptual development (Gori, 2015). Multisensory information is used in order to promote body perception that is a fundamental component of environmental knowledge related to movement and orientation in space (Koustriava and Papadopoulos, 2012) and might be impaired in congenitally blind subjects (Parreira et al., 2017). Moreover, relational activities with the therapist and the caregiver can have a fundamental role in our early re-habilitation, since emotional and relational aspects are strictly connected also to spatial cognition (Proulx et al., 2016), even though these aspects go beyond the scope of this article and have not been examined. The activities proposed in our intervention are intended to foster the development of spatial awareness, visuo-motor and visuo-cognitive abilities and learning skills, especially concerning geometry and mathematics. Multisensory experience is not only provided via specific re-habilitation activities in the clinical setting but also promoted through adaptations of the child’s everyday environments and activities, according to the International Classification of Functioning, Disability and Health approach (WHO, 2010). As neuropsychomotor development progresses, our re-habilitation work is enriched by the introduction of orientation and mobility training, aimed at promoting autonomy through functional exploration of space (Tinti et al., 2006). In this domain, an important milestone is the ability to switch from an egocentric to an allocentric frame of reference – the first regarding object location in reference to oneself, the second regarding an object location in reference to another object (Klatzky, 1998). Studies showed that visually impaired people tend to use an egocentric frame of reference, confirming the difficulties in the development of normal spatial cognition in blind people (Pasqualotto and Proulx, 2012; Ruggiero et al., 2018), which makes it mandatory an early training in this area (Fiehler et al., 2009; Fiehler and Rösler, 2010) that can be performed through the use of sensory modalities other than vision.

In conclusion, children with a congenital visual impairment can partially or completely lack a sensory experience that is essential for spatial development. Indeed, among sensory modalities, vision is the most pervasive one because it guides the maturation of the very first mental representations about space (Thinus-Blanc and Gaunet, 1997; Eimer, 2004; Pasqualotto and Proulx, 2012). Spatial events can be perceived in a syncretic or “gestaltic” way through vision, which allows the subject to acquire a whole series of information about their shape, dimensions, color, and contrast. Consequently, visual experience shapes the nature and the structure of space, motivating the infant to initiate exploratory activities in the surrounding environment (Kestenberg, 1979). For this reason, pieces of evidence suggest that the visual system has a central role in coordinating all the other perceptual-sensory systems and in guiding actions in space (Duffy, 1978; Fazzi et al., 2005a), raising questions about how to intervene on these aspects.

## Lessons Learned and Recommendations

In the last decades, there has been a raise of interest for visual disability, not only for what concerns enhancements and impairments in spatial knowledge due to the lack of visual experience but also for what concerns the development and the introduction of specific re-habilitation interventions to improve quality of life of visually impaired people. Nevertheless, there has been a simultaneous lack of studies assessing different rehabilitation approaches and outcome measures (Elsman et al., 2019). Moreover, standardized primary (e.g., regarding visual functioning or general perceptual skills) and secondary (e.g., functional status, quality of life, social, and working inclusion) outcome measures are currently not available for the visually impaired population. To our knowledge, only one study (Finocchietti et al., 2019) proposed a first possible goal standard test to evaluate spatial impairment in visually deprived children. In this sense, the use of technological devices could be extremely helpful for visually impaired children in order to reach rehabilitation goals, especially in the field of mobility and autonomy, as it has been shown in some recent works (Cappagli et al., 2017b, 2019).

## Conclusion

The case of M. demonstrated that an early multisensory and multidimensional re-habilitation can play an important role in the promotion of overall neuropsychomotor development in children with congenital visual impairment without Central Nervous System involvement. Spatial cognition development can particularly benefit from early activities proposed in an enriched environment promoting body knowledge, object permanence and space exploration through multisensory experience. Nevertheless, specific outcome measures, besides randomized controlled trials (RCTs), are needed to confirm our empirical and anecdotal evidences.

## Data Availability Statement

The datasets generated for this study are available on request to the corresponding author.

## Ethics Statement

Written informed consent was obtained from the patient’s parents for the publication of this case report, including any potentially identifiable images or data included in this article.

## Author Contributions

FM, GA, GC, and SS contributed to study conception, design, and manuscript writing. AL and FD are directly involved in the rehabilitation process, and contributed to data collection and manuscript writing. GC, MG, and SS contributed to critical manuscript revisions and final approval of the submitted version. All authors contributed to the article and approved the submitted version.

## Conflict of Interest

The authors declare that the research was conducted in the absence of any commercial or financial relationships that could be construed as a potential conflict of interest.
